# Zinc Deficiency Is Common in Several Psychiatric Disorders

**DOI:** 10.1371/journal.pone.0082793

**Published:** 2013-12-19

**Authors:** Ole Grønli, Jan Magnus Kvamme, Oddgeir Friborg, Rolf Wynn

**Affiliations:** 1 Department of Clinical Medicine, Faculty of Health Sciences, University of Tromsø, Tromsø, Norway; 2 Division of Addictions and Specialized Psychiatric Services, University Hospital of North Norway, Tromsø, Norway; 3 Department of Psychology, Faculty of Health Sciences, University of Tromsø, Tromsø, Norway; Chiba University Center for Forensic Mental Health, Japan

## Abstract

**Background:**

Mounting evidence suggests a link between low zinc levels and depression. There is, however, little knowledge about zinc levels in older persons with other psychiatric diagnoses. Therefore, we explore the zinc status of elderly patients suffering from a wide range of psychiatric disorders.

**Methods:**

Clinical data and blood samples for zinc analyzes were collected from 100 psychogeriatric patients over 64 of age. Psychiatric and cognitive symptoms were assessed using the Montgomery and Aasberg Depression Rating Scale, the Cornell Scale for Depression in Dementia, the Mini-Mental State Examination, the Clockdrawing Test, clinical interviews and a review of medical records. In addition, a diagnostic interview was conducted using the Mini International Neuropsychiatric Interview instrument. The prevalence of zinc deficiency in patients with depression was compared with the prevalence in patients without depression, and the prevalence in a control group of 882 older persons sampled from a population study.

**Results:**

There was a significant difference in zinc deficiency prevalence between the control group (14.4%) and the patient group (41.0%) (χ^2^ = 44.81, df = 1, p<0.001). In a logistic model with relevant predictors, zinc deficiency was positively associated with gender and with serum albumin level. The prevalence of zinc deficiency in the patient group was significantly higher in patients without depression (i.e. with other diagnoses) than in patients with depression as a main diagnosis or comorbid depression (χ^2^ = 4.36, df = 1, p = 0.037).

**Conclusions:**

Zinc deficiency is quite common among psychogeriatric patients and appears to be even more prominent in patients suffering from other psychiatric disorders than depression.

**Limitations:**

This study does not provide a clear answer as to whether the observed differences represent a causal relationship between zinc deficiency and psychiatric symptoms. The blood sample collection time points varied in both the control group and the patient group. No data regarding zinc supplementation were collected.

## Introduction

Zinc is a trace element that is essential for the optimal function of the human body, especially the brain. The highest concentrations of zinc in the brain are found in the hippocampus and amygdala regions [1]. Zinc is an important cofactor in more than 300 cellular enzymes influencing various organ functions [2]. Furthermore, a lack of zinc can lead to immune insufficiency, infection, diarrhea, skin eruptions and dermatitis [3].

Important sources of zinc include meat and fresh fish [4]. Zinc deficiency occurs in all age groups and nationalities [1]. A study conducted in five European countries revealed zinc deficiency in 31% of people over 60 years of age [4]. In addition, the study found significant differences between countries. Among hospitalized elderly individuals, a prevalence of 28% has been demonstrated [5].

Several studies have revealed a connection between low plasma zinc levels and depression [4], [6]–[8]. Animal studies have demonstrated that zinc deficiency in rodents enhances depression-like symptoms [9], [10]. In animals, depression-like symptoms that are related to zinc deficiency, appear to be reversed by antidepressant treatment [10], [11].

Several randomised controlled trials (RCT) have been conducted to explore the impact of zinc supplementation on depressive symptoms. Two trials have examined zinc supplementation as an adjunct to antidepressant drug treatment in patients with clinical depression. In a study by Nowac et al., 14 patients received an antidepressant and either placebo or zinc supplementation. Zinc supplementation significantly reduced scores on the Beck Depression Inventory (BDI) and the Hamilton Depression Rating Scale (HADRS) after 6 and 12 week of supplementation compared with placebo treatment [12]. This finding led to a larger RCT in which 60 patients received an antidepressant (imipramine) and either placebo or zinc supplementation. In this study, the researchers did not find a significant difference in the BDI or HADRS scores between adjunctive treatments with placebo or zinc supplementation [13]. However, in a subgroup of 21 patients with treatment-resistant depression, a significantly greater reduction in depression scores was exhibited by the group that received zinc compared to the placebo group. These trials were rather small, but the results have been supported by animal studies. Zinc deficiencies in rats and mice have been demonstrated to induce depressive symptoms, that are refractory to antidepressant treatment [11], [14].

Most studies of zinc deficiency have focused on depression. However, zinc has also been linked to a possible role in the pathogenesis of Alzheimer's disease (AD), but a causal role in AD has not yet been demonstrated [15]. There is no evidence of a difference in serum zinc concentration between patients with AD and controls [16]–[18]. Patients with dementia in a psychogeriatric population are characterized by psychiatric symptoms and behaviour disturbances, and we have not found any studies investigating zinc deficiency in this subgroup of demented patients. Furthermore, the relation between zinc and psychosis is unclear. However, to our knowledge, no studies have investigated zinc deficiency and zinc levels in older patients with psychosis.

Moreover, to our knowledge, no studies have compared the zinc status of a psychogeriatric population (exhibiting a broad range of psychiatric disorders) with that of healthy controls. It is important to examine whether zinc deficiency is common elderly patients with diagnoses other than depression. A better knowledge of zinc status in the elderly is important beyond the possible association between low zinc levels and psychiatric disorders. Low zinc levels are also associated with poor wound healing and an impaired immune response [19], [20]. Studies of nursing home residents, for example, suggest that low zinc levels may be a risk factor for pneumonia in the elderly [21].

### Aims of the present study

The aims of this investigation were 1) to compare the prevalence of zinc deficiency in patients referred to a psychogeriatric department and in a control group and 2) to compare the prevalence of zinc deficiency in patients with depression and in patients with other psychiatric diagnoses.

## Methods

### Study population

Patients older than 64 years who were referred to a psychiatric hospital in the northern part of Norway during the study period of March 2010 to December 2011 were eligible for inclusion in the study. The hospital covers a population of 255,000. We excluded patients with ongoing infections or who were not able to communicate due to their medical condition (i.e., severe dementia). In total 107 patients were asked to participate in the study, and five patients refused to participate. Two patients were excluded due to ongoing physical illness (infection). The participants were diagnosed using the structured Mini International Neuropsychiatric Interview (MINI+) [22], the Montgomery-Asberg Depression Rating Scale (MADRS) [23], the Mini Mental State Examination (MMSE) [24], the Clock-drawing Test [25], clinical interviews, observations, and medical records. Because of the medical conditions (e.g.,dementia), it was not possible to administer the MADRS to all patients: thus the Cornell Scale for Depression in Dementia [26] was used for 22 patients. Diagnoses were set according to ICD-10 criteria (WHO, 1992). Blood samples were drawn in the morning (before 10 AM), within the first three days of the stay and were analyzed for zinc and albumin. In addition, a wide range of other tests were performed, but not analyzed in the present study.

The control group included persons who were recruited from a population-based health study in Tromsø, a town with 70,000 inhabitants in northern Norway (The 6^th^ Tromsø Survey, 2007–2008). We restricted the analysis to participants older than 64 years. In total, 4017 men and women in this age group participated in the study, resulting in an overall participation rate of 66%. Non-fasting blood samples were drawn, and serum from all participants was frozen. Serum zinc and albumin from a random selection of 882 individuals were later analyzed and used for further comparison with the patient group.

### Zinc and albumin analyses

In both the patient group and the control group, venous blood samples were collected for the measurement of zinc and albumin. For the zinc analysis, trace-metal-free tubes and special gloves were used to avoid contamination. The samples were frozen and stored at −70 C. Serum zinc was subsequently analyzed using a flame atomic absorption at 213.9 nm (Perkin Elmer A-Analyst 800 Atomic Absorption Spectrophotometer).

In the patient group, blood samples were collected in the morning (before 10 AM). However, due to other considerations, 64% of the samples were collected after patients fasted overnight and 36% of the samples were collected under non-fasting conditions. In the control group, all samples were collected under non-fasting conditions between 8 AM and 12 AM. The International Zinc Nutrition Consultative Group (IZiNCG) [27] has recommended different serum zinc cut-off values depending on the patient's gender, fasting or non-fasting state, and time of measurement (i.e., AM or PM). We applied the cut-off values for zinc deficiency as defined in the IZiNCG guidelines (Table 1). A large proportion of zinc in serum is bound to albumin [28]. Therefore, we performed an additional assessment of serum albumin using the brom-cresol green method (Hitachi Modular P, Roche). The lower reference level for serum albumin was 34.0 g/L.

**Table 1:** Lower cut-off levels for zinc deficiency (µmol/L).

	Females	Males
Am fasting	10.7	11.3
Am non-fasting	10.1	10.7
PM	9.0	9.3

### Statistical analyses

For the statistical analysis, SPSS 20 (SPSS, Inc., Chicago, Illinois, USA) was used. Descriptive analyses were conducted to describe the characteristics of the sample, and the Kolmogorov-Smirnov test was used to examine assumptions of normal distributions. Due to a difference in the cut-off values in the fasting and non-fasting conditions, we based the statistical between group tests (patient vs. control) on a dichotomously defined zinc deficiency score (0-no deficiency, 1-deficiency). Differences in baseline characteristics between patients and controls were analyzed using Chi-square tests (dichotomous data) and independent samples t-tests (continuous data). Differences in the prevalence of zinc deficiency between different patient groups were analyzed with Chi-square tests. The association between zinc deficiency and the sample (patient vs. control) was expressed as an odds ratio (OR) with 95% confidence intervals (CI) from a logistic regression analysis. Differences in zinc levels across different patient groups were tested using one-way analysis of variance. A *p-*value <0.05 was accepted as statistically significant.

### Ethical considerations

All patients who were deemed candidates for participation were provided oral and written information about the study. For patients who were unable to provide consent alone due to their medical conditions, their next of kin were provided similar information. Patients and the next of kin (when relevant) provided written consent prior to inclusion in the study. Competency to provide consent was assessed according to previously established guidelines [29]. The Regional Medical Ethics Committee for North Norway (REK North) approved the current study.

## Results

The 100 psychogeriatric patients (62 females/38 male) included in the present study suffered from a range of disorders, including dementias, psychotic disorder, bipolar disorder, unipolar depressive disorder, and anxiety disorders. We found that 41 patients had a depressive disorder (mean MADRS = 25.3 (7.9)), as a first time depressive episode, recurrent depression or as part of a bipolar disorder. We also identified 20 patients who had depression comorbid with another diagnosis, such as dementia or organic mood disorder (mean MADRS = 19.9 (10.4)). In 39 of the patients, we did not identify any depressive disorders (mean MADRS = 4.9 (3.7)). In this group, 20 patients had dementia (10 cases of Alzheimer's disease), 11 patients had a psychotic disorder, and 8 patients had other non-depressive disorders. The diagnostic distribution in these three groups is presented in Table 2. The rate of antidepressant use in the three patient groups (depression, comorbid depression and other diagnoses) were, respectively, 76%, 75% and 25%.

**Table 2:** Diagnoses of patients.

Diagnoses	*N (male/female)*
Patients with depression as the main diagnosis	
First time depression	*6 (4/2)*
Recurrent depression	*27 (11/16)*
Bipolar depression	*8 (3/5)*
Patients with depression secondary to other diagnoses	
Alzheimer's dementia	*10 (4/6)*
Vascular dementia	*2 (1/1)*
Other organic mental disorders	*2 (1/1)*
Psychotic disorders	*1 (1/0)*
Anxiety disorders	*5 (1/4)*
Patients with no depressive symptoms	
Alzheimer's dementia	*10 (7/3)*
Vascular dementia	*4 (2/2)*
Other dementia	*6 (1/5)*
Other organic mental disorders	*3 (1/2)*
Psychotic disorders	*11 (2/9)*
Bipolar disorder, manic episode	*2 (0/2)*
Anxiety disorders	*2 (2/0)*
*Somatoform disorders*	*1 (0/1)*

### The prevalence of zinc deficiency

The prevalence of zinc deficiency in the psychogeriatric patient group was compared to the prevalence in a control group of 882 persons (444 females/438 males). Table 3 lists the characteristics of the patient group and the control group. In the patient group, 41.0% (41/100) had zinc deficiency, compared with 14.4% (127/882) in the control group. This difference was significant (n = 982, χ^2^_df = 1_ = 44.81, p<0.001). We found a non-significant difference in the prevalence of zinc deficiency between males (47.4%) and females (37.1%) in the patient group. In the control group, the prevalence of zinc deficiency was 18.5% in males and 10.4% in females, which was a significant difference (n = 882, χ^2^_df = 1_ = 11.83, p<0.001).

**Table 3:** Characteristics of the patient and control groups.

	Patients	Controls	*p-values*
Female/male (%)	62/38 (62.0/38.0)	444/438 (50.3/49.7)	p = 0.027*
Age (SD)	76.5 (7.2)	72.2 (5.7)	p<0.001**
Living alone (%)	53.0	37.1	p<0.001*
Smoking (%)	29.9	13.6	p<0.001*
BMI (SD)	25.3 (5.2)	27.0 (4.2)	p<0.001**
Albumin g/L (SD)	41.0 (3.4)	45.0 (2.3)	p<0.001**
Zinc deficiency–males (%)	47.4	18.5	p<0.001*
*Zinc deficiency-females (%)*	*37.1*	*10.4*	p<0.001*

*χ^2^ = Chi-square test, ** t =  Student's t-test. Notes.

The median zinc level in the non-fasting patient group (n = 36) was 10.7 µmol/L compared to 12.2 µmol/L in the control group (also non-fasting). The median zinc levels in the fasting patient group was 11.2 µmol/L. In the patient group, the prevalence of zinc deficiency was 42.2% in the fasting group and 38.9% in the non-fasting group, which was a not a significant difference.

The association between zinc deficiency and the patient or the control group status was further analyzed with logistic regression analyses. Zinc deficiency was associated with depression/comorbid depression and other psychiatric disorders after adjusting for the fasting condition, gender and age (Table 4). Both age and gender, but not the fasting condition, made a small contribution to the model (OR(gender) = 2.0 (1.4–2.8), p<0.001, OR(age) = 1.05 (1.0–1.1), p = 0.003). In a multivariate model that adjusted for age, gender, smoking status, living alone, body mass index (BMI), albumin and fasting condition, only gender (OR = 2.2 (1.5–3.2), p<0.001) and albumin (OR = 1.3 (1.2–1.4), p<0.001) made significant contributions to the model. The analyses without albumin are likely the most relevant, as we discuss below.

**Table 4:** Adjusted odds ratio (95% CI) for the association between zinc deficiency and the patient/control status.

Group	*Zinc deficiency, OR (95% CI)*	
	Adjusted for age, gender and fasting condition	*Multivariate adjusted* *
Control	1.0 (reference)	*1.0 (reference)*
Depression or comorbid depression	2.5 (1.1–5.5) p = 0.024	*1.0 (0.4*–*2.4) p = 0.95*
*Other psychiatric disorders*	*5.8 (2.3*–*14.4) p<0.001*	*2.4 (0.9*–*6.8) p = 0.09*

Adjusted for age, gender, BMI, smoking status, living alone, albumin levels and fasting condition.

### Zinc deficiency in relation to psychiatric diagnosis

We identified zinc deficiency in 41.0% of the psychogeriatric patients. The prevalence rates of zinc deficiency and zinc levels in the three main diagnostic groups are presented in Table 5. The difference in the prevalence of zinc deficiency between patients with depression as the main diagnosis and patients with other psychiatric diagnoses was not significant. However, when we compared all patients with depressive symptoms (main or comorbid depression) to patients with other psychiatric diagnoses, the prevalence was significantly higher in the latter group (Table 5). The difference in the prevalence of zinc deficiency between patients with depression as their main diagnosis and patients with depression as a comorbid diagnosis was not significant. These associations were not confounded by age, sex, smoking status, living alone, BMI, fasting/non-fasting state or albumin levels as these variables did not differ significantly between the three patient groups. The Pearson correlation between zinc levels and MADRS scores in the patient group was not significant. This was also the case when we separately analyzed the patient subgroups with depression as either main diagnosis or comorbid diagnosis. Among the patients with dementia (n = 32, including 20 with Alzheimer's disease), we identified zinc deficiency in 48.5% of the patients, but this prevalence was not significantly different from patients without dementia.

**Table 5:** Zinc levels and prevalence of zinc deficiency in the three diagnostic groups.

	Depression as the main diagnosis	Depression as co-morbid diagnosis	Other psychiatric diagnoses
Zinc deficiency (%)*	36.6	25.0	53.8
Zinc levels µmol/L(SD)**	11.4 (1.7)	11.7 (1.4)	10.7 (2.1)

χ^2^_df = 1_ = 4.36, p = 0.037). Significant difference in zinc deficiency between patient groups with depression/comorbid depression and other psychiatric diagnoses (

= 2.63, df = 2, p = 0.078). Distribution between fasting/non-fasting conditions was equal in the three patient groups. No significant differences between the three patient groups, using one-way analysis of variance (F

## Discussion

The major findings of this study were as follows: 1) There was a significantly higher prevalence of zinc deficiency in the psychogeriatric patients compared with the elderly controls. 2) The prevalence of zinc deficiency was significantly higher in patients without depression compared with patients with depression as the main or comorbid diagnosis. 3) There was no significant relationship between zinc levels and MADRS scores in patients with depression as the main or comorbid diagnosis.

### Zinc and psychiatric disorders

We found a high prevalence of zinc deficiency in the patient group compared with the control group. Several studies have demonstrated lower zinc levels in patients with depression than in healthy controls [6], [8], [30]. However, there is limited knowledge about zinc deficiency in patients with diagnoses other than depression. The current study found an even higher prevalence of zinc deficiency in patients with other psychiatric diagnoses than in patients with depression or comorbid depression. Dementia and psychosis accounted for the largest diagnostic groups among these patients. Zinc has been linked to a possible role in the pathogenesis of Alzheimer's disease (AD), but a causal role in AD has not yet been definitively demonstrated [15]. Research has mainly focused on abnormal zinc homeostasis, which may be involved in β-amyloid plaque formation [31], [32]. To the best of our knowledge, there is no established link between serum levels of zinc and the development of dementia. In one study, patients with AD were compared with patients who exhibited mild cognitive impairment and with normal controls, but no significant differences in the serum zinc concentrations were found [18]. This result is in line with other studies that revealed no significant differences in serum zinc levels between controls and AD patients [16], [17], [33]. However, the individuals with dementia in the current patient group presented severe behavioral disturbances or psychiatric symptoms (i.e., psychosis, depression, or anxiety), and were, therefore, not representative of “typical” demented patients. Notably, nearly 50% of the patients in this group had zinc deficiencies. Thus, one could speculate whether the lack of zinc contributes to behavioural problems and psychiatric symptoms in dementia. The group of patients with dementia was rather small and heterogeneous, so it is of course difficult to draw any firm conclusions. The significance of zinc in relation to psychosis is unclear. To our knowledge, no studies have investigated zinc levels in relation to psychosis in the elderly. Previous investigations have compared the zinc level of patients with schizophrenia to that of healthy controls. One study found no significant difference [34] whereas another study revealed a difference [35], thereby reflecting the diverging results found in previous studies [34].

Research in the field of zinc status and psychiatric disorders has primarily focused on depression. Several studies have found a connection between low zinc levels and depression [4], [6]–[8], but a few studies have not confirmed these findings [36], [37]. A main question is whether zinc deficiency actually contributes to the development of depression, or if zinc deficiency is simply caused by a change in diet due to the onset of depressive symptoms. Animal studies, however, suggest that zinc deficiency induces depression-like symptoms. When rodents are fed a zinc-deficient diet, they develop depression-like symptoms, including anorexia, anhedonia, increased anxiety behaviour, and increased periods of immobility, which have been reported in a number of studies [9], [10], [38]. Animal experiments have demonstrated that zinc can enhance the action of antidepressants in depressive models, such as the forced-swim test and the tail suspension test, and in chronic unpredictable stress [39]–[41]. This finding has also been supported by treatment studies in humans. Two RCTs have revealed potential benefits of zinc supplementation as an adjunct to conventional antidepressant drug therapy [12], [13].

In the present study, the serum zinc levels in patients with depression did not correlate with the MADRS scores. Although the findings might suggest that the magnitude of the serum zinc decline did not reflect the severity of the depression, other explanations for these results are plausible. The present findings correspond to similar results in two other studies [6], [8], but stand in contrast to those reported by Maes [30], who detected a statistically significant correlation between zinc levels and HADS scores. The first two studies [6], [8], included patients with treatment-resistant depression, whereas the latter study did not [30]. In addition, it has been proposed that the lack of a correlation between zinc levels and the severity of depression might be explained by the fact that all of the patients belong to a group of severely depressed individuals [8]. Patients who were included in the current study had a marked severity of depression, as they had been admitted to a psychiatric hospital. These subjects were also predominantly treatment-resistant, but we did not explore this aspect further.

To our knowledge, no previous studies have examined zinc deficiency across a broader range of psychiatric disorders among elderly people. The present findings are interesting because they suggest that zinc may play a role in a wide range of psychiatric disorders. Among patients with thyrotoxicosis, some individuals develop anxiety, others develop depression or psychosis, and still others, do not develop psychiatric disorders at all [42]. We find the same pattern in for example patients with hyperparathyroidism [43], [44] and in patients with low levels of vitamin B12 [45]. These results suggest that unknown individual factors may make certain people more vulnerable than others to developing psychiatric symptoms due to alterations in these hormones and micronutrients.

Several hypotheses may explain the relation between depression and zinc deficiency. Zinc can down regulate the glutamate response by inhibiting post-synaptic N-methyl-D-aspartate receptors (NMDA) [46], and is also involved in regulating brain-derived neurotrophic factor (BDNF) expression in the brain [47]. Reports have shown that zinc can increase the density of 5-HT1A and 5-HT2A serotonin receptors in the hippocampus and frontal cortex [48]. However, there is also mounting evidence that zinc plays an important role in the immune system and zinc deficiency has been reported to increase levels of pro-inflammatory cytokines [49]. This increase of pro-inflammatory cytokines also increases levels of the heavy-metal-binding protein metallothionein. Furthermore, it has been suggested that this mechanism contributes to the reduced zinc levels that are observed in patients with depression [50]. There is some evidence that alterations in the immune system may play a role in several types of psychiatric disorders [51], but it is unclear whether this factor might explain the current findings.

### Dietary intake of zinc

The present study did not include dietary data for the patient or control groups. Therefore, differences in the dietary intake of zinc might have contributed to the findings. Several studies have supported a connection between low dietary zinc and depression; however, no studies have investigated whether there is an association between low dietary zinc levels and a broader range of psychiatric disorders. A study of Europeans aged 60–84 years found a relationship between low dietary zinc intake, plasma zinc status and depressive symptoms [4]. A study of 402 postgraduate students in Malaysia, found a relationship between a low dietary intake of zinc and depression [52]. In one study, a low dietary zinc intake was positively associated with depression in women, but not in men [53]. A population study of habitual diets and mental health in women revealed an association between dietary zinc levels and depressive symptoms, but no such association with anxiety disorders [54]. These investigations do not present clear evidence for a causal relationship between low dietary zinc levels and depression; however, they support similar findings in animal studies. One could argue that psychiatric disorders are accompanied by decreased appetite and, therefore, a decreased overall dietary intake, including that of zinc. However, in the current investigation, BMI was not a significant predictor. Moreover, prior studies have not found an association between anorexia or weight loss and low zinc levels [30].

### Zinc and albumin

In the present study, the level of albumin was lower in the patient group than in the control group. In a logistic regression model, albumin and gender were significant predictors of zinc deficiency. In a model without albumin, belonging to the patient group was the strongest predictor. The relationship between zinc and albumin is complex. It has been argued that reduced albumin levels only partially explain a reduction in zinc levels [50]. There is increasing evidence that depression and other psychiatric disorders are accompanied by an activation of the inflammatory response system (IRS) [51], [55]. Activation of the IRS is known to lower the zinc level (due to increased production of metallothionein), but also lowers the albumin level [50]. Plasma zinc is primarily bound to albumin (70%) and to α2-macroglobulin (18%), with the remainder bound to other proteins and amino acids [56]. Only 1 of every 50 albumin molecules is bound to a zinc atom, and it has been argued that this fact renders it difficult to ascertain how a reduction in albumin could lead to significantly reduced zinc levels [28]. The IZInc group reported that only hypoalbuminemia (<34 g/L) may significantly influence zinc levels [27]. We conclude that the different levels of albumin in the current patient and control groups cannot fully explain the large difference in the prevalence of zinc deficiency. Therefore, analyses with only age, gender and fasting condition as confounders might provide the most interesting results (Table 4).

### Strengths and limitations

A major strength of this study was the inclusion of patients who suffered from different psychiatric disorders and the comparison of these individuals with a large group of controls. The diagnostic procedures that were applied to the patient group were extensive. In addition to diagnostic instruments, clinical observations and data from medical records were used. Another strength of the study was that we controlled for confounding factors, including age, gender, smoking habits, BMI, albumin levels, and whether the patient was living alone or with another individual. These factors have allowed us to make interesting observations regarding the zinc levels in elderly psychiatric patients and in elderly controls.

Although we do not believe that zinc supplementation is common among the elderly in our uptake area, we lacked data on zinc supplement intake in the control group. We also lacked data regarding psychiatric symptoms in the control group. However, this group was quite large and unlikely to include a substantial number of persons with severe psychiatric illness. In addition, the blood-sampling time point differed in the two groups. Zinc levels tend to decrease slightly during the day, as reflected in Table 1. This fact might have increased the control group's (for which the blood sampling lasted until 12 AM) risk of being categorized as zinc deficient. Nevertheless, this situation would strengthen the present findings. The prevalence of zinc deficiency was similar in the fasting and non-fasting patient groups. We also controlled for the fasting/non-fasting condition (using a logistic regression analysis) and this factor did not appear to influence the main results.

## Conclusions

In this study, we found that zinc deficiency is quite common among psychogeriatric patients and appears to be even more prominent in patients without depression. However, the present investigation does not provide a clear answer as to whether the observed differences represent a causal relationship between zinc deficiency and psychiatric symptoms. Although the causes of the differences in zinc deficiency rates are unknown, we propose several possible explanations. Differences in albumin levels may explain a smaller part of the differences in zinc levels. Another factor might be differences in the dietary intake of zinc. Prior studies have suggested potential differences in the dietary intake of zinc between depressed patients and controls. This factor might also apply to patients with other psychiatric disorders compared with controls. Increased levels of inflammatory factors might also contribute to lower levels of zinc in depressed patients; however, it is unknown whether this could also be a factor in patients with other psychiatric disorders. Although there might be different causes of low zinc levels, animal studies [40], [41] and intervention studies [57] have suggested that the administration of zinc to depressed patients zinc may yield a positive effect. The etiology of psychiatric disorders is complex, and biological, environmental, medical and possibly modifiable risk factors may all play a role [58]. Additional research on this topic is necessary to further explore the role of zinc in the treatment of depression and to further investigate whether zinc may be of importance to other psychiatric disorders.
